# Construct validity of the Experiences of Therapy Questionnaire (ETQ)

**DOI:** 10.1186/s12888-014-0369-6

**Published:** 2014-12-31

**Authors:** Gordon Parker, Amelia Paterson, Kathryn Fletcher, Georgia McClure, Michael Berk

**Affiliations:** School of Psychiatry, University of New South Wales, Sydney, Australia; Black Dog Institute, Sydney, Australia; Department of Clinical and Biomedical Sciences, University of Melbourne, Victoria, Australia; Orygen Research Centre, Melbourne, VIC Australia; Mental Health Research Institute, Melbourne, VIC Australia

**Keywords:** Therapy, Psychotherapy, Measure, Validity, Side-effects, Psychology, Questionnaire

## Abstract

**Background:**

The Experiences of Therapy Questionnaire (ETQ) is a reliable measure of adverse effects associated with psychotherapy. The measure has not been subject to validity analyses. This study sought to examine the validity of the ETQ by comparison against a measure of therapist satisfaction.

**Methods:**

Participants were recruited from the Black Dog Institute’s website and completed all measures online, at two time points (two weeks apart). Correlational analyses compared scale scores on the ETQ with related constructs of the Therapist Satisfaction Scale (TSS). To exclude any impact of current depression on ratings, we examined correlations between salient ETQ and TSS scales after controlling for depression severity.

**Results:**

Forty-six participants completed all the measures at both time points. Hypothesised associations between the ETQ and TSS scales were supported, irrespective of current depression severity.

**Conclusions:**

The validity of the ETQ is supported; however limitations of the study are noted, including generalizability due to sample characteristics.

## Background

We previously proposed [1] that psychotherapy can, as for any other active therapy, risk possible ‘side-effects’. Such a proposition risks being viewed as incongruous for a ‘talking therapy’ and elicited some engagement with correspondents when we originally published our proposition [2-4]. Some were positive comments, in stating that ‘all clinicians need to acknowledge that any treatment that has the capacity to greatly help the patient can also in equal measure have the potential to cause harm’ [3] or in basically agreeing with the premise [4]. However, correspondents also urged prudence when distinguishing between negative effects of therapy and negative effects of the therapist [3], and cautioned about framing an event such as sexual exploitation of a patient as a ‘side-effect’ instead of a gross violation of conduct [2,3]. Such views encourage consideration of what is really meant by the term ‘side-effect’. For drug therapies, the term is normally used for any adverse event that is considered to be a negative consequence of the medication. In psychotherapy, side-effects have previously been described to include ‘adverse treatment reactions’ that are defined as unwanted events caused by treatment which has been applied according to the rules [5] thereby distinguishing these side-effects from therapist misconduct. We suggest, in accordance with the above definition, that reasons for terminating drug therapy or psychotherapy due to negative experiences (e.g. feeling judged by a therapist) can both equally be termed side-effects. We understand that this may be considered a controversial definition but note that the term ‘side-effect’ has been used to describe a range of negative outcomes of psychotherapy from deterioration in functioning [6], lowered expectations of therapeutic benefit [6] to harmful therapy techniques [7]. Indeed, it has been argued that training therapists to evaluate side-effects of their therapy is crucial and standardized instruments to evaluate such effects are recommended [5,6]. In addition to this, it has been suggested that the best avenue of obtaining information on both the positive and negative effects of psychotherapy is via client feedback [7,8].

Thus, we define a psychotherapeutic ‘side-effect’ broadly - reflecting an adverse outcome as a consequence of therapy. Feedback from our initial paper was taken into consideration when we developed (Parker et al., 2013) a self-report measure to assess negative outcomes in psychotherapy - the Experiences of Therapy Questionnaire (ETQ) – and where we also established the reliability of the ETQ [9].

Content validity was established during the development of the ETQ. First, studies assessing psychotherapeutic ingredients that impact on psychotherapy were reviewed. Items derived from this review process largely captured eight domains including client efficacy, the therapeutic relationship, impact of therapy, rational/treatment ‘fit’, treatment as restorative, therapist factors and the therapeutic setting. The items were reviewed by three clinicians (two psychiatrists and a psychologist) to ensure key aspects were represented. This process yielded 103 items that were then refined to the 63 contained in the final version of the ETQ [9]. The reliability of the ETQ has been reported elsewhere [9].

A factorial analysis favoured a five-factor solution. Factor one (‘Negative Therapist’), captures a construct where the therapist lacks empathy or respect, is intrusive in manner, or judgemental. Factor two (‘Preoccupying Therapy’) captures therapy which becomes consuming or overwhelming to the patient. Low scores on Factor three (‘Beneficial Therapy’) capture therapy being viewed as unsuccessful in addressing reasons bringing the individual to seek treatment. Factor four (‘Idealisation of Therapist’) applies when the patient becomes dependent on or even physically attracted to the therapist. Factor five (‘Passive Therapist’) captures a therapy style of doing little or using techniques not believed in by the patient.

As the construct validity of the ETQ has not yet been considered, we undertook the current investigation. We sought to establish the construct validity of the ETQ by examining levels of agreement between the ETQ and a conceptually related instrument assessing therapy satisfaction - The Therapist Satisfaction Scale (TSS).

## Methods

### Data collection

Participants were recruited via The Black Dog Institute Website. The Black Dog Institute is a Sydney-based organisation that provides clinical intervention and management, and undertakes research, professional training and public education dedicated to improving the lives of individuals affected by mood disorders. The Institute website offers information, online tools and resources for both mental health professionals and the community. In 2012, 846,585 unique viewers accessed the Black Dog website. The website contains links to current research projects, and this was the method of recruitment employed in the current study. To be included in the study, participants had to be over 18 years of age and have proficient English language skills. Participation was restricted to those who had received therapy previously, and excluded those currently receiving therapy. Website respondents viewed an information and consent form and interested viewers were invited to respond anonymously in a study asking ‘Have you had an interesting experience in therapy?’- in essence a neutral invitation avoiding the risk of limiting respondents to only those who had experienced a negative therapy. Participants were asked to indicate what type of therapy they had received and were given access to therapy descriptions. Participants were also asked for relevant information regarding their therapy experience (length of therapy, session number, session frequency, problem under treatment) and demographic information including age, sex and if on medication for the problem targeted in therapy’.

### Measures

Participants were required to complete all measures on two occasions (two weeks apart) as the broader study was designed to assess test re-test reliability [9]. Participants were emailed the measures two weeks following the initial completion, and sent one reminder to maximise completion response rate at time two.

The ETQ is a 63-item scale. Participants are asked to rate statements on a 5-point Likert scale (ranging from strongly disagree (1), strongly agree (5)) assessing how each statement reflects their therapeutic experience. The five factors of the ETQ have been found to demonstrate good internal consistency (.67-.96) and high test-retest reliability (0.76-0.96) [9]. Scale scores are derived by summing raw scores of each individual item that loads on that scale, including reverse scoring for negative loaded items. Twenty one items load on ‘Negative Therapist’ scale, 13 items on the ‘Preoccupying Therapy’ scale, 12 items on the ‘Beneficial Therapy’ scale, 6 items on the ‘Idealization of Therapist’ scale and 11 items on the ‘Passive Therapist’ scale. A higher mean score indicates a higher degree of endorsement for that scale (i.e. higher mean score for ‘Negative Therapist’ indicates a negative impression of the therapist).

The TSS is a measure of therapeutic alliance and should, theoretically, be related to subscales of the ETQ. The TSS measures therapist characteristics that contribute to patient satisfaction. It is a 47-item scale, rated on a 4-point Likert scale (very true (1), very untrue (4)), and derives 5 scales of: care-concern, directive-control, critical-confronting, communication and charisma. All scales have been shown to have high internal reliability (.79-.96) [10]. Scale scores are derived by summing raw scores of each individual items that loads on that scale, including revers scoring for negative loaded items. Ten items load on the ‘Care-Concern’ scale, 9 items on the ‘Directive-Control’ scale, 11 items on the ‘Communication’ scale, 8 items on the ‘Charisma’ scale and 9 items on the ‘Critical-Confronting’ scale. A higher mean score indicates a higher degree of endorsement for that scale (i.e. a higher mean score for ‘Care-Concern’ indicates the therapist was perceieved as empathic and understanding by the patient).

The Quick Inventory of Depressive Symptoms- Self Rated (QIDS-SR) is a 16-item scale measuring severity of depression over the past week. Participants are asked to select which item best reflects how they have been feeling over the past seven days on a 4-point Likert-scale. The measure has been found to have high internal consistency (.86) [11] and high construct validity [12].

### Hypotheses testing

Correlational analyses were undertaken to examine associations between ETQ and TSS scale scores to determine construct validity, both before and after controlling for depression severity by use of the QIDs-SR. As the TSS ‘Critical Confronting’ scale relates to a judgemental therapist, but also one who increases tension during therapy and is similar to the ETQ ‘Negative Therapist’ scale, we anticipated that these two scales would have a strong positive association. The TSS ‘Care and Concern’ scale captures empathy and understanding, and the TSS ‘Communication’ scale captures open and realistic interactions - both of which are key ingredients of successful therapy and we therefore predicted these scales would highly correlate with the ETQ ‘Beneficial Therapy’ scale. The TSS ‘Charisma’ scale captures the extent to which the patient finds the therapist special and unique, and conceptually links with the ETQ ‘Idealisation of Therapist’ scale, so that we predicted these scales to be highly correlated. The TSS ‘Directive Control’ scale relates to the therapist exerting direct influence or providing specific guidance to the patient and would be expected to have a high inverse association to the ETQ ‘Passive Therapist’ scale. The ETQ ‘Preoccupying Therapy’ scale was not anticipated to be associated with any of the TSS scales and was examined in an exploratory manner.

As depression severity might artefactually influence a patient’s ratings of their therapist and therapeutic experience, current depression severity was quantified by the QIDs-SR. It was expected that depression severity would have a moderate, positive correlation with the ETQ ‘Negative Therapist’ and ‘Passive Therapist’ scales. It was also expected that depression severity would have a moderate, inverse association with the ETQ ‘Beneficial Therapy’ scale.

## Results

Of the 106 participants completing the first administration of the questionnaires, 46 completed the second administration with an average interval of 19 days (SD = 7.1 days). The 46 participants were older than the 106 (t = −2.3, p = 0.02) and were less likely to be taking medication for their problem while receiving therapy (χ^2^ = 4.0 p = 0.04), however they did not differ in length of therapy (χ^2^ = 5.2), total number of sessions (t = −1.0), frequency of therapy (χ^2^ = 4.7), gender (χ^2^ = 0.17), problem under treatment (χ^2^ = 5.3), or type of therapy (χ^2^ = 4.4) (all p > 0.05). These 46 comprise our study sample, and their characteristics are provided in Table 1.

**Table 1:** **Sample Characteristics n = 46**

Gender:	Male	5 (10.9%)
	Female	41 (89.1%)
Therapist:	Psychologist	28 (60.9%)
	Psychiatrist	11 (23.9%)
	Counsellor	3 (6.5%)
	Social Worker	2 (4.3%)
	GP	2 (4.3%)
Therapy:	General Counselling	12 (26.1%)
	CBT	18 (39.1%)
	IPT	4 (8.7%)
	Psychodynamic Psychotherapy	6 (13.0%)
	Long-term psychotherapy	6 (13.0%)
Principal problem:	Anxiety	5 (10.9%)
	PTSD	5 (10.9%)
	Depression	22 (47.8%)
	Bipolar	5 (10.9%)
	Eating Disorder	3 (6.5%)
	Other	6 (13.0%)
Frequency of therapy	< Once per month	3 (6.5%)
	Once per month	10 (21.7%)
	Once every 3 weeks	5 (10.9%)
	Once per fortnight	11 (23.9%)
	Once per week	14 (30.4%)
	Twice a week	1 (2.2%)
	> Twice per week	2 (4.3%)
Duration of therapy	< One month	1 (2.2%)
	Two-three months	9 (19.6%)
	Four to six months	7 (15.2%)
	Six to twelve months	5 (10.9%)
	One to two years	10 (21.7%)
	More than 2 years	14 (30.4%)
Medication	Yes	26 (56.5%)
	No	20 (43.5%)
Total sessions	Mean	76.5
	SD	202.4
	Range	1-1200
	Median	16

Table 2 reports the means and standard deviations of the ETQ and TSS scales. Table 3 reports Pearson correlation analyses of the ETQ and TSS measures. As both measures were administered at two differing time-points, correlations for both time-points are reported. Significant correlations were quantified between the relevant ETQ and TSS scales as hypothesised. Correlations were interpreted using Dancey and Reidy’s categorisation where ±1 indicates a perfect correlation; ±0.7 - ±0.9 indicates a strong or high correlation; ±0.4 - ±0.6 indicates a moderate correlation; ±0.1 - ±0.3 indicates a low or weak correlation and 0 indicates no correlation [13]. Partial correlations were used in order to control for depression severity, and no associations differed in significance or strength when controlling for depression severity.

**Table 2:** **Means of ETQ and TSS Subscales (n = 46)**

**Measure**	**Subscale**	**Mean (SD)**
ETQ scales:	Negative Therapist	44.09 (20.6)
	Preoccupying Therapy	36.96 (9.7)
	Beneficial Therapy	39.65 (13.3)
	Idealisation of Therapist	12.33 (3.6)
	Passive Therapist	27.9 (10.2)
TSS scales	Critical Confronting	15.78 (6.5)
	Care and Concern	29.48 (8.1)
	Charisma	17.26 (4.3)
	Communication	34.28 (6.0)
	Directive Control	25.54 (4.8)

The ETQ and TSS scale scores are derived by the summing of raw scores of the individual items that load on that construct, including reverse scoring for negative loaded items. A higher mean score indicates a higher degree of endorsement for that scale.

**Table 3:** **Correlational analyses examining a priori associations between ETQ and TSS scales with partial correlations controlling for depression severity using the QIDS (n = 46)**

**ETQ scales**	**TSS scales**	**Time point**	**Correlation**	**Partial correlation**
‘Negative Therapist’	‘Critical Confronting’	1	0.77**	0.79**
		2	0.82**	0.81**
‘Preoccupying Therapy’	‘Critical Confronting’	1	0.53**	0.54**
		2	0.57**	0.53**
	‘Care and Concern’	1	−0.51**	−0.48**
		2	−0.54**	−0.55**
	‘Charisma’	1	−0.26	−0.24
		2	−0.26	−0.34*
	‘Communication’	1	−0.50**	−0.46*
		2	−0.47**	−0.48**
	‘Directive Control’	1	−0.48**	−0.46*
		2	−0.52**	−0.46*
‘Beneficial Therapy’	‘Care Concern’	1	0.78**	0.80**
		2	0.78**	0.78**
‘Beneficial Therapy’	‘Communication’	1	0.73**	0.73**
		2	0.74**	0.72**
‘Idealisation of Therapist’	‘Charisma’	1	0.57**	0.59**
		2	0.49**	0.50**
‘Passive Therapist’	‘Directive Control’	1	−0.46**	−0.46*
		2	−0.54**	−0.54**

**Significant p ≤ 0.001, *Significant P < 0.05.

## Discussion

Establishing the validity of a new scale is fundamental to its use. This study was designed to determine whether the validity of the ETQ could be supported, with encouraging findings. The ETQ ‘Negative Therapist’ scale was highly correlated with the TSS ‘Critical Confronting’ scale, with such correlations observed at both administration time-points so indicating stability. Similar results were observed for associations between the ETQ ‘Beneficial Therapy’ and the TSS ‘Care and Concern’ and ‘Communication’ scales. The ETQ ‘Idealisation of Therapist’ scale was expected to be associated with the TSS ‘Charisma’ scale, but the correlation was only moderate. It appears that while the TSS ‘Charisma’ scale and the ETQ ‘Idealisation of Therapist’ scale have come conceptual overlap, the ETQ ‘Idealisation of Therapist’ scale may reflect more inherent negativity (e.g. “I didn’t feel like I was capable of making decisions without first consulting my therapist”), in contrast to the more positive characteristics implied by the TSS ‘Charisma’ scale (my therapist “Was the sort of person people would look up to and respect”). Nonetheless, the association was stable over time. It was anticipated that the final ‘Passive Therapist’ scale would be negatively associated with the TSS ‘Directive Control’ scale. While the negative correlations were stable, the association was only moderate, and possibly reflect the breadth of items covered under ‘Passive Therapist’.

Correlational analyses involving the ETQ ‘Preoccupying Therapy’ scale showed it to be significantly positively correlated with the TSS ‘Critical Confronting’ scale and negatively correlated with the ‘Directive Control’, ‘Care and Concern’ and ‘Communication’ TSS scales. The positive association with the TSS ‘Critical Confronting’ scale (which includes items such as the therapist was ‘critical of me’) reflects how therapy with a practitioner who seems to do nothing but criticise could lead to rumination - as seen in ‘Preoccupying Therapy’ items such as ‘with therapy I am aware of having more problems than before therapy’. Similarly, therapy that is low on ‘Care and Concern’ and ‘Communication’ would also become preoccupying as the patient is likely to feel invalidated and unheard by their therapist. The negative association with ‘Directive Control’ appears to imply that without some direction to the therapy it can become preoccupying, and possible stagnant.

It was surprising that depression severity did not affect the significance or strength of any of the observed associations between the ETQ and TSS. This may reflect the robustness of these domains of therapeutic outcomes regardless of disorder severity, and possibly disorder type.

Having a reliable and valid measure of psychotherapy ‘side-effects’ has potential advantages, most clearly in studies evaluating comparative therapies but where findings may well be distinctly influenced by therapy not just therapist factors. In the development paper, participants were asked if they left therapy for reasons other than their problem improving. One-quarter reported they left as their problem was not improving, 17% indicated that they were dissatisfied with their therapist or the therapy and 8% stated that their therapy was actually making them worse [9]. It is unlikely that these patients would ever have discussed their therapy termination with their practitioner especially if they felt the therapy was making them worse. In such cases the ETQ could be a useful mechanism for uncovering termination reasons which might otherwise have gone undisclosed. We therefore consider that the ETQ could be utilized in research to help distinguish unhelpful, or beneficial, aspects of different therapy modalities. For the ETQ to be considered appropriate in this research context, the questionnaire would also need to demonstrate the ability to detect clinically important change over time [14]. External responsiveness, the extent to which changes in a measure over time relate to changes in a reference measure of health [15], could be assessed in future studies by comparing responses on the ETQ to an overall measure of mental health status.

Study results further quantify the psychometric properties of the ETQ, establishing the measure as a valid indicator of psychotherapy ‘side-effects’. Our findings also raise a semantic issue. The ETQ has a premise of quantifying ‘side-effects’ while the TSS seeks to measure ‘treatment alliance’, the latter being a softer and less provocative construct, though in reality the two domains have considerable overlap. Failure to establish a treatment alliance can reflect a range of factors brought by the patient, the therapist or both, and which do not ascribe ‘cause’ or ‘blame’ to the therapist of necessity. The use of ‘side-effects’ is a more active descriptor that implicates the therapist or therapy more directly, albeit effectively impugning no contribution by the patient to the process. Each has their own connotations and each variably risks being too lenient or too condemnatory in their tone and positioning. In addition, there is considerable overlap between certain scales of the ETQ and TSS as demonstrated by the large associations found in this paper. We note that despite the semantic difference between ‘therapeutic alliance’ and ‘side-effects’, it may be redundant to administer both instruments in their entirety in all research circumstances. Progress may be assisted by studies that use both measures in, addition to qualitative information, to determine which of these measures may be most useful in different circumstances.

This study does have limitations. Results were derived from a small sample and it is possible that more reminders may have increased completion of the measures at the second time point. We also did not collect information such as time since the therapy ceased, gender of the therapist or the type of medication participants used. The sample obtained via the internet was likely to over-represent patients being treated for mood disorders due to recruitment through the Black Dog Institute website and was also skewed towards females and those receiving long-term therapy. It is therefore possible that females and those seeking longer term therapy were more likely to actively seek information from the Black Dog website and be exposed to research recruitment invitations. In addition, no pilot testing or cognitive assessments were completed with participants to assess understanding of the measures. It is also possible that, by inviting participants who ‘have had an interesting or unusual experience of therapy,’ we may have attracted individuals who have had extreme or unusual experiences of psychotherapy (both positive and negative). As such the generalisability of these findings should be taken with some caution but, as our main objective was a validation study, such limitations might be expected to have had minimal impact on those analyses.

## Conclusions

This study supports the validity of the ETQ as a measure of negative ingredients or side-effects in psychotherapy. The ETQ can be used to determine potential causes of therapy failure, and as such may be of particular use in treatment efficacy studies, and might be used alone or to complement measures of therapeutic alliance.

## Abbreviations

ETQ: Experiences of therapy questionnaire
TSS: Therapist satisfaction scale
QIDS: Quick Inventory of depressive symptoms – self rated
